# Intramolecular Interaction with the E6 Region Stabilizes the Closed Conformation of the N-SH2 Domain and Concurs with the Self-Inhibitory Docking in Downregulating the Activity of the SHP2 Tyrosine Phosphatase: A Molecular Dynamics Study

**DOI:** 10.3390/ijms23094794

**Published:** 2022-04-27

**Authors:** Emanuele Bellacchio

**Affiliations:** Area di Ricerca Genetica e Malattie Rare, Bambino Gesù Children’s Hospital, IRCCS, Piazza S. Onofrio 4, 00165 Rome, Italy; Emanuele1B@yahoo.it

**Keywords:** tyrosine phosphatase, tandem SH2 domain, enzyme regulation, protein–protein interactions, molecular dynamics, allostery

## Abstract

The localization and activity of the SHP2 tyrosine phosphatase across different cellular compartments to the target substrates are steered by the binding of phosphotyrosine (pY) peptides to the tandem SH2 domains. The most N-terminal domain (N-SH2) can also keep the enzyme inactive by intramolecular occlusion of the catalytic site. Enzyme activity can be recovered by an allosteric disruption of this self-inhibitory docking upon the binding of pY peptides to the N-SH2 domain. Prior to this, the N-SH2 domain must abandon the closed conformation because it impedes the access of pY peptides to the binding cleft. Although it cooperates with the self-inhibitory docking in the negative regulation of the phosphatase activity, the structural determinants of the stability of the closed conformation in the self-inhibited phosphatase are still elusive. To address this issue, a molecular dynamics simulation study is carried out. It is shown that the closed conformation is stabilized by the interaction of the N-SH2 domain with a conserved peptide portion in the region encoded by *PTPN11* exon 6 (E6).

## 1. Introduction

Protein tyrosine phosphorylation modulates many protein features, such as stability, subcellular localization, patterns of molecular recognition, activity, and other functions contributing to signaling and biological regulation [1]. In concert with tyrosine kinases, which add a phosphate group, the protein phosphorylation status is controlled by protein tyrosine phosphatases, which catalyze the removal of phosphate groups via a cysteinyl-phosphate intermediate [2]. The Src homology 2 (SH2) domain containing phosphatase 2 (SHP2, also known as tyrosine-protein phosphatase non-receptor type 11) is encoded by the *PTPN11* gene and characterized by two SH2 domains arranged in tandem (N-SH2, a.a. 1–103, and C-SH2, a.a. 111–213) N-terminally to the phosphatase domain (PTP, a.a. 253–529). SHP2 has functions ranging across the regulation of cell growth, tissue inflammation, cell survival in oxidative stress pathways [3], hematopoiesis and differentiation [4,5], cell proliferation [6], and migration [7].

Mutations in the *PTPN11* gene causing a loss of phosphatase activity are associated with LEOPARD syndrome [8], while those enhancing the activity are linked to Noonan syndrome [9]. Furthermore, mutations incrementing the phosphatase activity are common in juvenile myelomonocytic leukemia [10], and this led to classifying *PTPN11* as an oncogene. Mutations enhancing the enzyme activity are also found in some human solid tumors (e.g., lung cancer, colon cancer, neuroblastoma, and melanoma) and adult acute myelogenous leukemia [11]. The oncogenic role of *PTPN11* was also supported by the finding that leukemia-associated mutations prolong cell survival [12] and cause defects in erythroid differentiation [13]. Consequently, SHP2 attracted interest as a molecular target for inhibition in anti-cancer therapy [14,15,16]. To further illustrate the complex roles of this tyrosine phosphatase in diseases, a tumor suppressor function has also emerged [17]. There is also growing evidence that SHP2 can contribute to autoimmune disorders after links with this enzyme have been found in systemic lupus erythematosus [18], autoimmune liver disease [19], and autoimmune encephalomyelitis [20].

While measurable down- or upregulation of the phosphatase activity helps to assign a pathogenic role to SHP2 variants, the clinical importance of other variants might remain unclear if the affected biochemical properties of the phosphatase can be revealed only by specialized analysis, such as the specificity or affinity for pY peptides and the sensitivity to the pY peptide levels necessary to trigger the activation of the enzyme or to drive its transport to specific cellular compartments. Consequently, in order to fully ascertain the pathogenicity and phenotypes, researchers have deemed it necessary to understand how the different SHP2 variants can affect the several properties of SHP2 [21,22] and how these alter the complex network of this phosphatase.

SHP2 can enter in low basal phosphatase activity by intramolecularly inserting the D’E loop (residues 58–62 of the N-SH2 domain) into the catalytic pocket of the phosphatase domain [23]. Full activity is unleashed upon undocking from the PTP domain, which is triggered by the binding of pY peptides to the N-SH2 domain. Since two non-overlapping regions of N-SH2 are exploited for the interactions with the pY peptide and the PTP domain, N-SH2 undocking from PTP occurs allosterically [24]. Crystallographic studies of the isolated N-SH2 complexed with pY peptides [25,26] revealed that these ligands bind this domain between the EF loop (residues 66–68) and BG loop (residues 89–92). Critical for the ability to bind a pY peptide is the separation presented by the BG and EF loops, which can vary, thus regulating the access of these ligands into the N-SH2 domain’s binding cleft. In particular, in the open conformation, the two loops are adequately distant to allow the entry of pY ligands, as can be observed in the crystal structures of the isolated N-SH2 domain with bound pY peptides. On the other hand, in the closed conformation, the two loops are too close for ligand binding, as determined in the crystal structures of the self-inhibited SHP2 tyrosine phosphatase. Interestingly, the isolated N-SH2 also presents the open conformation when crystallized without pY peptides [25], suggesting this is the most stable conformation of the domain in the absence of intramolecular interactions with the rest of the protein. However, the bound and unbound forms of the isolated N-SH2 domain might differ in the restraints caused by the binding of pY peptides that reduce the domain’s plasticity and ability to gain full shape complementarity for its docking onto the phosphatase domain [27].

The SH2 domains in tandem enhance the versatility of the SHP2 phosphatase by efficiently conveying the substrates to the catalytic domain, recruiting the enzyme into microdomains hosting substrates [28,29,30], enhancing substrate specificity, functioning as an adapter, and binding with high-affinity bisphosphoryl tyrosine-based activation motifs [31,32,33,34]. Furthermore, the organization in tandem has been proposed to assist the smooth sliding of N-SH2 over C-SH2 back and forth from the PTP domain for efficient timing of self-inhibition and activation by the enzyme [35].

**Figure 1:**
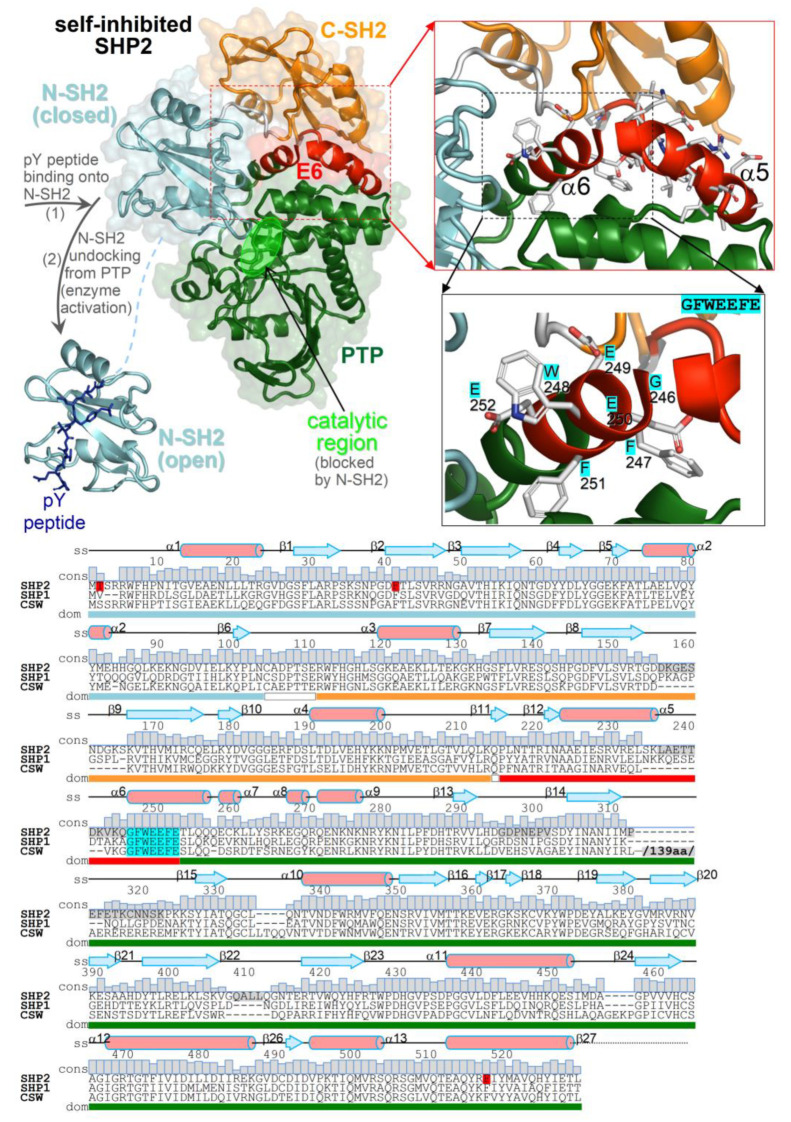
Structure of SHP2 and multiple-sequence alignment of tyrosine phosphatases characterized by the same tandem SH2 domain architecture (SHP2, SHP1, and corkscrew). (**top**) Crystal structures of the self-inhibited SHP2 (Protein Data Bank (PDB) 2SHP) and the isolated N-SH2 domain of SHP2 bound to a pY peptide (PDB 1AYA). The domains are in distinct colors; E6 is in red, and inter-domain linkers are in white. The close-up view highlights E6 and the invariant 246-GFWEEFE-255 segment in more detail. (**bottom**) Sequence alignment of SHP2, SHP1, and corkscrew with secondary structure elements and amino acid conservation (displayed on top of residues), with ranges of domains and inter-domain linkers (colored bars below the alignment). Mutated and disordered residues in the SHP2 crystal structure are highlighted in red and gray, respectively. The 139 amino acid-long insertion in the corkscrew sequence is omitted (its position is indicated).

The aim of this study was to understand what the determinants of the closed conformation preference by the N-SH2 domain are when this is docked onto the phosphatase domain. Its synchronism with self-inhibition suggests that the closed conformation might be stabilized by N-SH2 interactions with extra-domain regions. Specifically, these regions could be the phosphatase domain or the exon 6-encoded peptide (E6, amino acids 215–252) as both make physical contact with N-SH2 in the self-inhibited phosphatase, but neither of them interact with the isolated form of N-SH2 (Figure 1). Despite a structured portion of E6 also being packed onto the PTP domain, it does not seem necessary for catalysis, since substrate dephosphorylation still occurs even without most E6 residues, as shown by the SHP2 constructs formed by residues 230–593 [36] and residues 246–547 [37]. E6 conservation among tyrosine phosphatases featuring tandem SH2 domains (SHP1, SHP2, and corkscrew) would suggest importance for this region, but its function remains elusive. To examine whether E6 has a role in the closed conformation, molecular dynamics (MD) simulations were carried out on the N-SH2 domain in different structural contexts: isolated N-SH2 (N-SH2_closed_, closed conformation; N-SH2_open_, open conformation), wild-type self-inhibited SHP2 (SHP2-wt), self-inhibited SHP2 carrying mutations weakening the interaction of E6 with N-SH2 (SHP2-W248A and SHP2-W248A/E252A), and self-inhibited SHP2 fully deprived of this interaction (E6 deletion construct, SHP2-ΔE6).

## 2. Results

### 2.1. MD Simulation Stability

The stabilities of the MD simulations were assessed by monitoring the root mean square deviation (RMSD) at the backbone atoms. All simulated systems (SHP2-wt, SHP2-ΔE6, SHP2-W248A, SHP2-W248A/E252A, N-SH2_closed_, and N-SH2_open_) achieved a plateau within about 3 ns without drifts (Figure S1).

### 2.2. Interactions of E6 with N-SH2, C-SH2, and PTP in the Wild-Type Self-Inhibited SHP2

**Figure 2:**
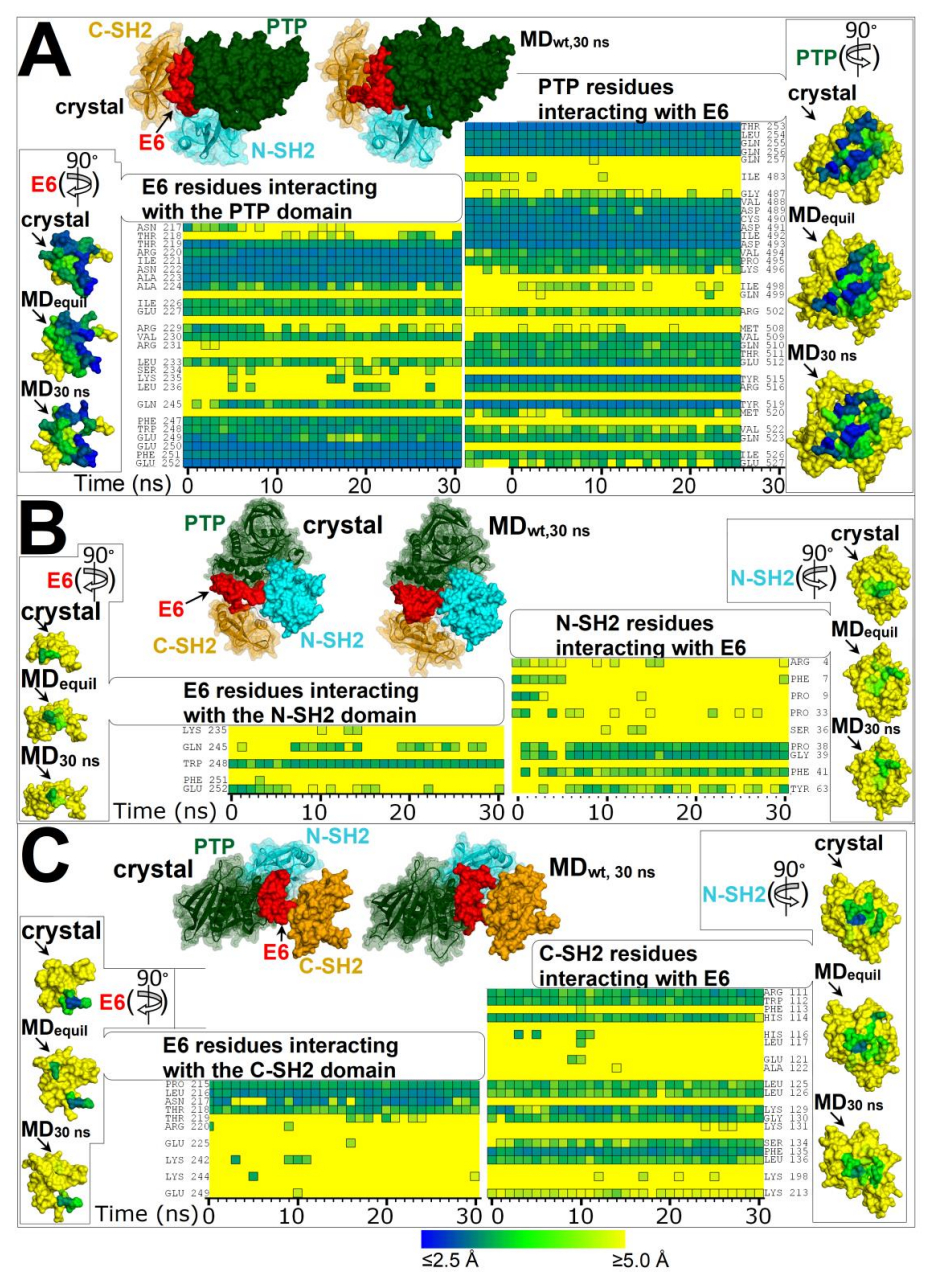
Residues forming the interfaces of E6 with domains PTP, N-SH2, and C-SH2 in the crystal structure of the self-inhibited SHP2 (PDB 2SHP) and at time intervals of the MD simulation of SHP2-wt. Heat maps representing the distances of E6 residues from each domain of SHP2 and vice versa calculated from conformers of the MD simulation (simulation time in the abscissa; time = 0 represents the distances calculated in SHP2 crystal structure). The color key for distances is shown at the bottom of the figure. (**A**) Residues at the interface between E6 and PTP. (**B**) Residues at the interface between E6 and N-SH2. (**C**) Residues at the interface between E6 and C-SH2. On top of each heat map is shown the self-inhibited SHP2 crystal structure and SHP2-wt at 30 ns of the MD simulation, both oriented for clear view of E6 binding to the various domains. Interacting residues are listed next to each heat map. At the side of each heat map are shown the molecular surfaces of E6 or the domains isolated from the SHP2 crystal structure and from the MD snapshots of SHP2-wt at 3 ns (ca. the time to achieve equilibration as assessed from RMSDs) and at 30 ns. These surfaces are colored according to the shortest distance from the interacting partner (E6 or a domain), employing the same color key as for the heat maps. The surfaces with mapped distances are rotated (as indicated by arrows) with respect to the parent multi-domain structure at the top of each figure section to obtain frontal view of relevant interacting residues.

In the self-inhibited enzyme, E6 interacts with all three domains of the SHP2 phosphatase (Figure 1). The residues involved in the interactions of E6 with each domain in the SHP2 crystal structure and at various times of the MD simulation of SHP2-wt are shown in Figure 2 (the individual names of the interacting residues are indicated in correspondence with each heat map row). The most extended interface of interaction with E6 involved the PTP domain, while only a few E6 residues interacted with either N-SH2 or C-SH2. In particular, during the simulation, only one E6 residue at the E6/N-SH2 interface, Trp248, presented stable interaction with N-SH2, while other residues interacted transiently (Lys235, Gln245, Phe251, and Glu252). In the N-SH2 domain, most of the residues interacting with E6 were located in the loop formed by residues 34–40.

### 2.3. Domain–Domain Interactions in the Self-Inhibited SHP2: Comparison of Wild-Type and E6 Deletion Constructs

To determine the importance of the E6 region in the various domain–domain interactions in the self-inhibited enzyme, the MD simulations of SHP2-wt and SHP2-ΔE6 were compared to identify possible differences in the inter-domain interfaces.

#### 2.3.1. Interactions between the N-SH2 and C-SH2 Domains

The N-SH2 and C-SH2 domains are held together by a short linker and few non-covalent interactions (dominated by the salt bridge between Arg5 and Asp192). No major changes in the N-SH2/C-SH2 interface were observed in the MD simulations of SHP2-wt nor in the SHP2-ΔE6 construct (Figure S2).

#### 2.3.2. Interactions between the C-SH2 and PTP Domains

The C-SH2/PTP interface did not change appreciably in the MD simulations of SHP2-wt but increased in extension in the simulation of SHP2-ΔE6 (Figure S3). This is an obvious consequence of the deletion of E6, which in the wild-type protein lays between C-SH2 and PTP and physically separates the two domains. This suggests that one role of E6 could be to prevent C-SH2 from collapsing on the PTP domain, which might abnormally hinder functional regions.

#### 2.3.3. Interactions between the N-SH2 and PTP Domains

The largest inter-domain interface in the self-inhibited SHP2 is the one formed by N-SH2 and PTP. Despite E6 appearing to collate the two domains, thus stabilizing the inactive enzyme configuration, the N-SH2/PTP interface did not differ appreciably in the SHP2-wt or SHP2-ΔE6 constructs (Figure S4), nor were there significant changes in the N-SH2/PTP area of contact (Figure S5) within the time length of these simulations.

### 2.4. Exploration of Constructs Presenting N-SH2 in a Different Structural Context

The synchronism with the self-inhibitory docking suggests that the closed conformation could be induced by some intramolecular interactions of N-SH2 with E6 or PTP, as no contact with these extra-domain regions can take place in the isolated N-SH2 domain presenting stable open conformation. To obtain insights into the determinants of the stability of the closed conformation, N-SH2 constructs presenting various combinations of closed or open conformations and different interactions of the 34–40 and D’E loops with E6 and PTP were investigated by MD simulations (the N-SH2 domain’s different structural contexts are summarized in Table 1).

### 2.5. RMSDs of the N-SH2 Domain

The RMSDs of the N-SH2 domain in the self-inhibited constructs (SHP2-wt, SHP2-ΔE6, SHP2-W248A, and SHP2-W248A/E252A) and in the isolated N-SH2 domain (N-SH2_closed_ and N-SH2_open_) did not differ significantly if calculated using the structures kickstarting the respective MD simulations as references (Figure S1). However, by recalculating the RMSDs of N-SH2 of SHP2-wt and SHP2-ΔE6 relative to the crystal structure of N-SH2 in open conformation (PDB 1AYA), an important decrement in the atomic displacements was seen in the E6 deletion construct but not in the wild-type SHP2 construct, and the source of this decrease could be identified in the loops formed by residues 34–40 and residues 65–69 (Figure S6). The change in the RMSDs of the 34–40 loop is consequential to the interaction of the N-SH2 region with E6, which can occur in the self-inhibited SHP2 but not in the E6 deletion construct. Quite surprisingly, the RMSD change was localized in amino acids 65–69 despite they did not interact with E6. The latter region is functionally important as it encompasses the EF loop functional in the closed or open conformation of the N-SH2 domain. When comparing SHP2-wt and SHP2-ΔE6, no difference could be seen in the RMSDs of the other two regions deemed important for N-SH2 functions, the BG loop (also determining the closed or open conformation), and the D’E loop (inhibiting the phosphatase catalytic pocket). The decreased RMSDs in SHP2-ΔE6 could suggest that this construct gained structural similarity with the crystal structure of the open conformation N-SH2 domain.

### 2.6. Volumes of the Groove in the pY Peptide-Binding Region of the N-SH2 Domain

**Figure 3:**
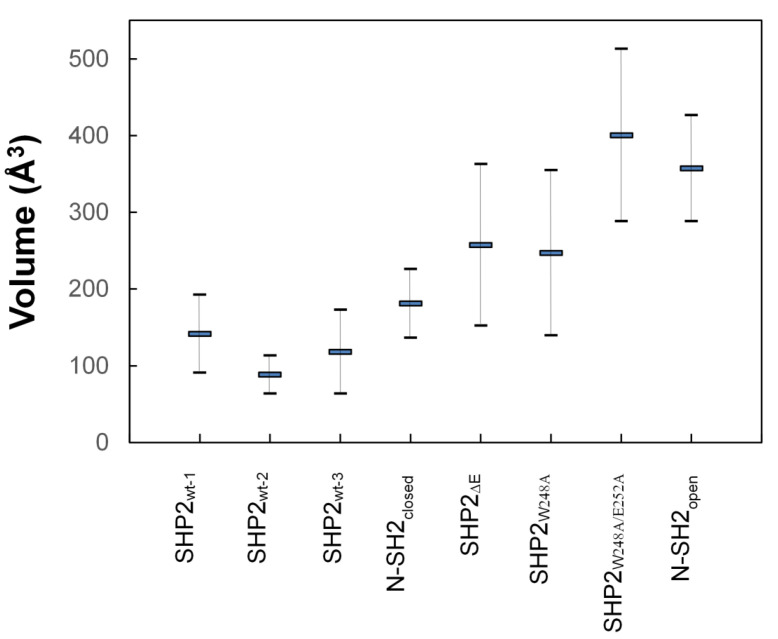
Average volumes of the groove in the pY peptide-binding region of the N-SH2 domain calculated in the MD simulations of SHP2-wt (triplicate MD runs 1–3), SHP2-ΔE6, SHP2-W248A, SHP2-W248A/E252A, N-SH2_closed_, and N-SH2_open_ (from snapshots collected every 1 ns in the 3–30 ns time interval).

To assess whether the deletion of E6 might indeed favor the change from closed to open conformation in the self-inhibited enzyme, the volume of the groove in the pY peptide-binding region of the N-SH2 domain was calculated and averaged from MD simulation snapshots. An increase in the volume could be observed in SHP2-ΔE6 compared with the three distinct simulations of SHP2-wt (Figure 3), indicating that the E6 deletion construct enlarged the space available between the BG and EF loops. It must be underscored that the modeled construct carrying such an important deletion was not intended to reproduce what would be the fold of the real protein construct. The SHP2-ΔE6 model was rather designed to examine how the N-SH2 domain would behave after the “sudden” disappearance of its interactions with E6 starting from the multi-domain protein prearranged in its native, self-inhibited configuration. As a matter of fact, incremented volumes were also observed in the self-inhibited structures carrying the much simpler single- and double-missense mutations W248A and W248A/E252A. As can be seen in the heat maps of Figure 2, Trp248 and Glu252 were E6 residues both contributing to the interactions with the N-SH2 domain such that their replacement with an alanine likely weakened such interactions. Comparing the SHP2-W248A and SHP2-W248A/E252A mutants, the latter had the most increased volume. This suggests that the two missense mutations, by cumulatively weakening the E6/N-SH2 interaction, can synergistically induce enlargement of the space used for pY peptide binding, which became similar to the open conformation structure N-SH2_open_. The latter maintained its large initial volume, which is characteristic of the open conformation. Coherence with the conformation of their parent structures was also exhibited by SHP2-wt and N-SH2_closed_, both preserving the small volume typical of the closed conformation. The statistical significance of an outcome in which all three simulations of the wild-type self-inhibited structure maintained the closed conformation and the simulations of all three mutated self-inhibited structures exhibited closed-to-open conversion was 0.05 according to the hypergeometric test. Taken together, these results indicate that the interaction of E6 with the N-SH2 domain has a role in the stability of the closed conformation in the self-inhibited SHP2 tyrosine phosphatase.

### 2.7. Critical Regions and Interactions in N-SH2 Determining the Conformation of the Domain

#### 2.7.1. State of the Art

**Figure 4:**
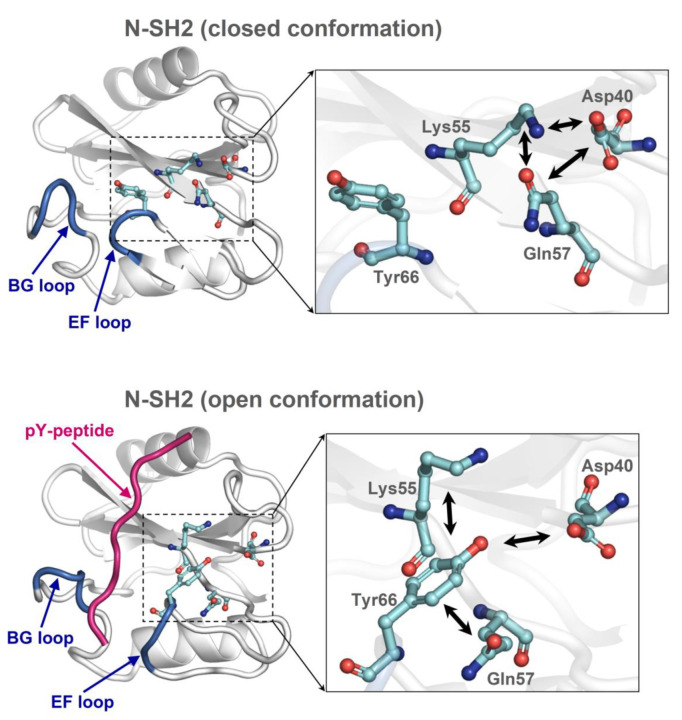
Closed and open conformations of the N-SH2 domain of the SHP2 tyrosine phosphatase. (**top**) Closed conformation (N-SH2 domain isolated from PDB 2SHP). (**bottom**) Open conformation with a bound pY peptide (PDB 1AYA). Shown is the Tyr66 conformational switch and the other critical residues Asp40, Lys55, and Gln57, whose mutual interactions (indicated by double arrows) determine the closed or open conformation, as highlighted by Guvench et al. [38].

Guvench et al. [38] discovered a key role of Tyr66 in the regulation of the EF loop functional conformations, determining that the open conformation is settled when the tyrosyl group is pulled away from the pY peptide-binding cleft. This is achieved by a set of side chain–side chain non-bonding interactions involving Tyr66: hydrogen bonding with Asp40, either hydrogen bonding or hydrophobic interaction with Lys55, and either hydrogen bonding or pi stacking with Gln57 (Figure 4). Conversely, mutual interactions among Asp40, Lys55, and Gln57 set free the tyrosyl group allowing the N-SH2 domain to regain the closed conformation. It is still unknown what drives Asp40, Lys55, and Gln57 rearrangement in favor of their mutually interacting configuration. It would be valuable to know the mechanism given the importance of the closed conformation. As a matter of facts, this conformation disfavors the recruitment of pY ligands by N-SH2 and delays undocking of this domain from PTP, thus cooperating with self-inhibition to negatively regulate the enzyme activity. It is also puzzling that the open and closed conformations are both thermodynamically stable, as evidenced by crystallographic and MD simulation studies. It would rather be expected that the enzyme could rapidly swap between the active and inactive states. This would ensure responsiveness to sudden changes in pY ligand types and concentrations in the diverse cellular microenvironments and physiological conditions encountered by the enzyme. As a matter of fact, for such a pleiotropic enzyme, it is necessary to process substrates as well as to avoid indiscriminate dephosphorylations in a timely manner. After all, abnormally high or low phosphatase activity both characterize a number of disease-associated PTPN11 mutations.

#### 2.7.2. The Separation of the BG/EF Loop during the MD Simulations of the N-SH2 Domain in a Different Structural Context

**Figure 5:**
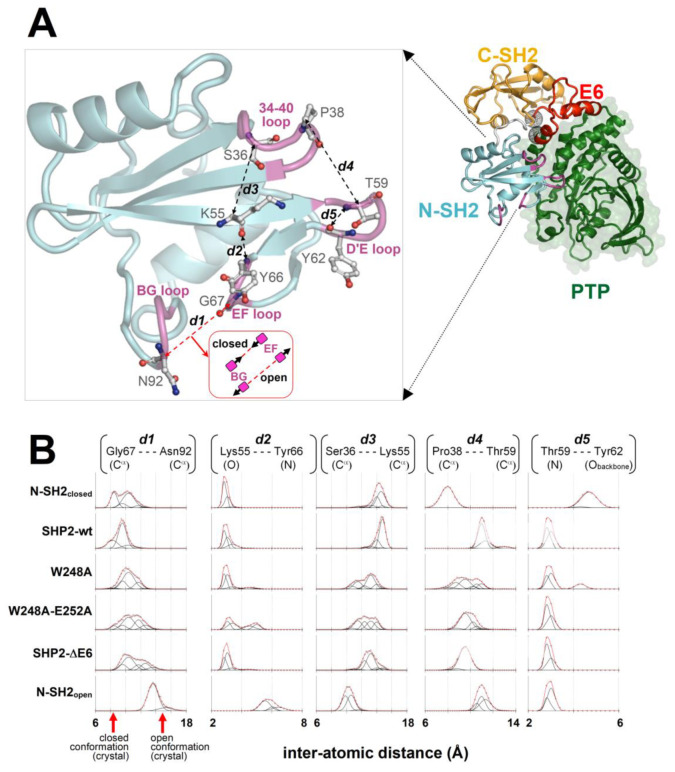
Conformational dynamics of key residues and loops of the N-SH2 domain. (**A**) Self-inhibited SHP2-wt structure (N-SH2 domain loops are colored in magenta, and the extremities of the E6 region are highlighted by the black spherical meshes centered at the C^α^ atoms of Gln214 and Thr253). In the enlarged view, the interatomic distances (dashed lines) between pairs of N-SH2 atoms highlighting critical loop/loop or residue/loop separations (discussed in the text) are shown. (**B**) Plots of the fraction of MD conformers in function of the interatomic distances traced in the N-SH2 domain above (obtained with this domain in different structural contexts; see text and Table 1). Pro38(C^α^)-Thr59(C^α^) indicates the separation of 34–40 and the D’E loop. Thr59(N)-Tyr62(O_backbone_) distance is reported to infer potential backbone-backbone hydrogen bonding between these two residues in the D’E loop. Gly67(C^α^)-Asn92(C^α^) represents the separation of the EF and BG loops, which determines the closed or open conformation (the arrows on the horizontal axis indicate the separations measured in the crystal structures of SHP2: closed conformation = PDB 2SHP; open conformation = PDB 1AYA). Lys55(O)-Tyr66(N) indicates the separation of Lys55 from the EF loop, also allowing one to infer potential backbone-backbone hydrogen bonding between the two residues. Ser36(C^α^)-Lys55(C^α^) measures the separation of the 34–40 loop from Lys55. The points (open circles) in the plots were obtained by calculating the fraction of MD conformers (recorded every 5000 femtoseconds in the 3–30-ns interval of simulations) with interatomic distances falling within a 0.15-nm long sliding window. Deconvolutions of the plots with Gaussian functions (black lines) and their sums (red line) are shown. The fitting was made by introducing a set of Gaussians (an identical number of Gaussians within a column of plots) and seeking squared minimization (globally for all plots in a column). In this procedure, the amplitude was allowed to vary independently for all Gaussians in all plots in a column, and their widths and centers were also varied independently within single plots but synchronizing these changes in the corresponding Gaussians of all other plots in the same column. With these constraints, the populations of the conformational ensembles characterized by particular loop/loop or residue/loop separations could be compared among the various MD simulations through the amplitudes of corresponding Gaussians in the same column of plots.

**Figure 6:**
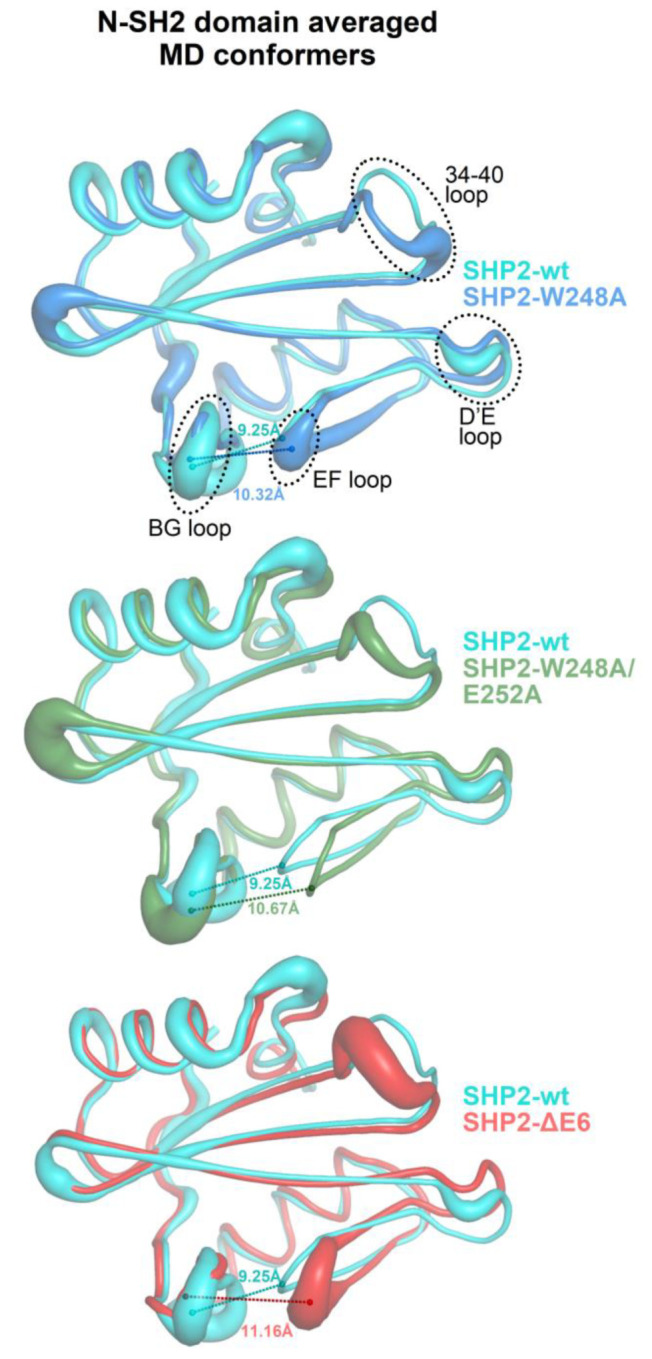
Averaged MD conformers of the N-SH2 domain from the simulations of the self-inhibited SHP2 and its mutants with weakened or annulled interactions between E6 and N-SH2 (averages were made on the conformers, collected every 5000 femtoseconds in the 3–30-ns time interval and previously aligned across N-SH2 domain residues 3–103 to the structures kickstarting the respective MD simulations). A larger ribbon thickness indicates a higher RMSD (calculated at C^α^ atoms). For the sake of clarity, pairwise structural superpositions between the wild-type SHP2-wt protein and each mutant (SHP2-W248A, SHP2-W248A/E252A, and SHP2-ΔE6) are presented. The distance between the EF and BG loops (measured between the C^α^ atoms of Gly67 and Asn92) is labeled on each averaged structure.

The plots of the fractions of the MD conformers versus the separation of the BG and EF loops and fitting with Gaussian functions allowed to identify a few conformational ensembles characterized by specific loop separations (Figure 5). In the case of SHP2-wt and N-SH2_closed_, the most populated ensembles presented BG/EF loop separations within 8.3 and 10.4 Å. The minimum value of this range was very close to the 8.2 Å measured for the N-SH2 crystal structure in a closed conformation (PDB code 2SHP), while the maximum value was only ca. 2 Å above this. The isolated N-SH2 construct, N-SH2_open_, presented only two main ensembles of conformers characterized by much larger BG/EF loop separations centered at 13.6 and 15.3 Å, respectively, which were close to the 14.8-Å long separation observed in the N-SH2 crystal structure in the open conformation (PDB 1AYA). Thus, the BG/EF loop separations measured during the MD simulations of SHP2-wt, N-SH2_closed_, and N-SH2_open_ were all consistent with the separations seen in the respective parent crystal structures. This implies that in these constructs, the N-SH2 domain maintained its initial conformation, whether it was closed or open. A different trend was shown by the self-inhibited constructs carrying the single- and double-missense mutations weakening the interaction of E6 with N-SH2 (SHP2-W248A and SHP2-W248A/E252A) or the E6 deletion (SHP2-ΔE6) fully abolishing this interaction. In fact, despite originating from the same self-inhibited crystal structure of SHP2, the simulation of the three mutants exhibited additional ensembles with longer BG/EF loop separations centered at 11.7, 12.6, and 13.6 Å. In particular, the greatest value was quite close to the 14.8-Å distance of the BG and EF loops in the crystallographic N-SH2 domain in open conformation. Among the three mutants, the ensembles exhibiting greater BG/EF separations became more populated the more the E6/N-SH2 interactions vanished (SHP2-W248A < SHP2-W248A/E252A < SHP2-ΔE6 (Figure 5)). This BG/EF separation trend paralleled the changes in volume in the pY peptide-binding region of N-SH2 shown in Figure 3, indicating conservation of the closed conformation by SHP2-wt and N-SH2_closed_, conservation of the open conformation by N-SH2_open_, and an increased propensity of the three mutants in undergoing closed-to-open conformation conversion (in the order of SHP2-W248A < SHP2-ΔE6 < SHP2-W248A/E252A). The opening of the BG and EF loops in the mutants can also be noticed in the averaged structures of the N-SH2 MD conformers (Figure 6). Taking into account the different N-SH2 structural contexts examined in this study (Table 1), these results highlight that the closed conformation, stable in the self-inhibited enzyme, could switch with ease to the open conformation upon weakening or abolishing the interactions of E6 with the N-SH2 domain.

#### 2.7.3. The Role of the Cation–pi Interaction between Lys55 and Tyr66

**Figure 7:**
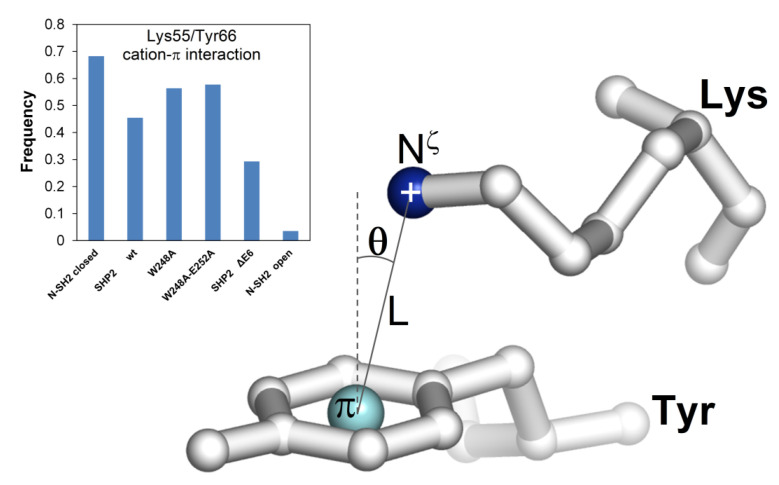
Fractions of MD conformers showing cation–pi interaction between Lys55 and Tyr66. The geometric conditions to identify this interaction were L ≤ 7 Å and θ ≤ 60° (see Materials and Methods for further details).

A finding emerging from this study is that the N-SH2 closed conformation was stabilized by interactions with E6. Since the EF loop was relatively distant from the N-SH2/E6 interface, this effect should be mediated allosterically. Guvench et al. [38] determined that the critical EF loop residue Tyr66 acts as a conformational switch, which can select the open conformation by undergoing side chain–side chain interactions with Asp40, Lys55, and Gln57. In the present MD study, two additional and distinct interactions also involving Tyr66 and Lys55 were found. One was hydrogen bonding of Lys55 oxygen with Tyr66 nitrogen, which occurred in all closed conformation constructs but not in the open conformation N-SH2 (see Lys55-Tyr66 distance plot in Figure 5) and thus appearing to be in competition with the above-mentioned interactions stabilizing the open conformation. The second was the Lys55/Tyr66 cation–pi interaction, which was observed with a comparable frequency in N-SH2_closed_, SHP2-wt, SHP2-W248A, and SHP2-W248A/E252A, less frequently in SHP2-ΔE6, and more rarely in N-SH2_open_ (Figure 7). Since the cation–pi interaction can exert attractive forces at longer distances than the other non-bonding interactions mutually attracting Lys55 and Tyr66, it might serve to enhance the ability of the two residues to join together after they are separated by the domain’s conformational changes. In particular, the Lys55/Tyr66 cation–pi interaction can aid the inter-conversion between closed and open conformations in both directions. In fact, in the closed conformation, the Tyr66 and Lys55 side chains were quite distant (Figure 4), and thus the cation–pi interaction was the first to mediate the mutual attraction of the two residues. Thereafter, the tyrosyl group could eventually shift to the alternative short-range interactions, with Lys55 also engaging Asp40, and Gln57 for full stabilization of the open conformation. On the other hand, after unloading the pY peptides, the N-SH2 domain might linger in the open conformation, owing to the stabilizing short-range interactions of Tyr66 with Asp40, Lys55, and Gln57. In this circumstance, the Lys55/Tyr66 cation–pi interaction can competitively step in and drive the domain into the closed conformation. Thus, by exchanging the type of non-bonding intra-domain interactions with Tyr66, other critical N-SH2 residues might support smooth conversions between the two functional conformations of the domain. To remark on the importance of this cation–pi interaction, cationic and aromatic residues at positions homologous to Lys55/Tyr66 are also present in the other phosphatases characterized by tandem SH2 domains, such as SHP1 and corkscrew.

## 3. Discussion

Guvench et al. [38] reported that Tyr66 determines the closed conformation, owing to intra-domain interactions of the tyrosyl group with the side chains of the Asp40, Lys55, and Gln57 triad (Figure 4). Given the importance of Tyr66 as a closed or open conformational switch, it is necessary to understand how the configuration of this tyrosine could be influenced by the somehow distant interactions between E6 and N-SH2. The triad of residues interacting with Tyr66 can undergo mutual interactions, and one of them, Asp40, is part of the 34–40 loop. Thus, the configuration of the Asp40, Lys55, and Gln57 triad has a direct dependance on the conformation of the 34–40 loop, which can change upon interaction with the extra-domain regions of the phosphatase. Insights were obtained by analyzing how this loop moves relative to the critical residues in the MD simulations of the constructs presenting the N-SH2 domain in different structural contexts. In the cases of SHP2-wt and N-SH2_open_, the distance of the 34–40 loop from the D’E loop was similar to that observed in the respective parent crystal structures (Figure 5). In SHP2-wt, this was due to the restraints imposed on the two loops by their contact with E6 and PTP. In this case, the 34–40 loop protruded outward from the N-SH2 domain surface to gain favorable interactions with E6. However, when the interaction with E6 was weakened or annulled, as in SHP2-W248A, SHP2-W248A/E252A, and SHP2-ΔE6, the 34–40 loop bent inward, achieving a stabilizing intra-domain contact with Lys55. This was evidenced by the decreased distance between the C^α^ atoms of Ser36 (residue in the 34–40 loop) and Lys55, which was observed in all three mutants (Figure 5). The bending of the 34–40 loop caused the relocation of Asp40 and the twisting of the β-strand containing Lys55 and Gln57. As a result, Asp40, Lys55 and Gln57 were brought closer to the Tyr66 tyrosyl group. Thus, weakening or annulling the interaction of E6 with N-SH2 in the self-inhibited enzyme had the final effect to bring the three residues into the configuration that stabilized the open conformation that was described by Guvench et al. [38]. This particular configuration was stable in the MD simulation of SH2_open_. Weakening or abrogating E6/N-SH2 interactions also caused the 34–40 loop to relax onto the D’E loop, as observed in SHP2-W248A, SHP2-W248A/E252A, and SHP2-ΔE6 (see the decreased distances between the C^α^ atoms of Pro38 and Thr59 in Figure 5). With respect to the separation of the D’E and 34–40 loops, a striking difference could be observed in the simulations of the two isolated N-SH2 domain constructs. In N-SH2_open_ the 34–40 loop maintained a constant distance from the D’E loop, whereas in N-SH2_closed_ the two loops became much closer. SH2_closed_ was also the only structure that lost the hydrogen bond between the Thr59 nitrogen and Tyr62 backbone oxygen (highlighted by the sudden increase in the Thr59-Tyr62 distance in Figure 5). This conferred the D’E loop with a peculiar conformation among all constructs. The unusual behavior of the D’E loop in N-SH2_closed_ might be an artifact arising from the atypical structural context of this domain construct. In fact, N-SH2_closed_ was obtained by computational truncation of the self-inhibited SHP2-wt, which produced an isolated N-SH2 domain yet was conformationally frozen as if it was still bound to PTP and E6. Instead, in the undocking path of the real enzyme, it can be thought that while the N-SH2 domain gradually reduces its contact with the extra-domain regions PTP and E6, its conformation likely undergoes stepwise adaptations. Thus, when interpreting the MD simulation of N-SH2_closed_, it must be taken into account that the construct does not represent an N-SH2 domain in full native conditions. It can be deduced that the stability of the closed conformation in the self-inhibited enzyme is not an intrinsic property of the N-SH2 domain but the consequence of its intramolecular interactions with the extra domain region E6. In particular, Tyr66 conformation is allosterically modulated by the interaction of E6 with the 34–40 loop.

## 4. Materials and Methods

### 4.1. Molecular Dynamics Simulations

The structures employed in the MD simulations were obtained as follows. The self-inhibited SHP2 protein (SHP2-wt, amino acid range: 1–529) was represented by the crystal structure of the phosphatase (PDB code 2SHP, chain A). The mutated residues T2K, F41L, F517S, and Q530L were restored to the wild-type ones. The missing residues (a.a. 1 and loops 156–160, 236–245, 295–301, 313–323, and 408–411) were constructed one at time with MODELLER (v. 9v8) [39].

The E6 deletion construct (SHP2-ΔE6) was obtained by removing residues 215–252 from SHP2-wt, followed by conjugation of the deletion boundaries Gln214 and Thr253. Since these two residues were proximal in the crystal structure, their conjugation required applying geometry optimization to them plus a few flanking residues (a.a. 212–214 and 253–255) and to the side chain of Arg111 that lays between Gln214 and Thr253 (Figure S7). The SHP2-W248A and SHP2-W248A/E252A mutants were obtained from SHP2-wt by introducing the single- and double-missense mutations W248A and W248A/E252A. The isolated N-SH2 domain in the closed conformation (N-SH2_closed_, a.a. 3–103) was derived by truncation from SHP2-wt. The isolated N-SH2 domain in the open conformation (N-SH2_open_, a.a. 3–103) was represented by the crystal structure of the N-SH2 domain of SHP2 (PDB structure 1AYA, chain A).

The molecular structures were embedded in explicit water solvent (TIP3P water model) with Na^+^ and Cl^−^ ions added to achieve electroneutrality and an ionic strength of 0.1 mol/L. Before the MD simulations, each molecular system was subjected to two cycles of minimization, equilibration, and minimization. In the first cycle, all protein atoms were restrained, while the water and ions were allowed to move freely. In the second cycle, only the protein backbone atoms were restrained (in these cycles, minimizations and equilibrations were carried out for 20,000 and 500,000 steps, respectively). Finally, all restraints were removed, and the systems were minimized again for 20,000 steps. The MD simulations were performed with NAMD (v.2.8) [40,41], employing the CHARMM22 protein force field [42] including CMAP correction under periodic boundary conditions using an integration step of 1 femtosecond and flexible bonds at a temperature T = 310 K, controlled with a Langevin thermostat, and using a damping coefficient of 1 ps^−1^. Short-range interactions were computed at every time step, and long-range electrostatic interactions were computer every two time steps, employing a switching distance of 10 Angstroms, cutoff of 12 Angstroms, and pair list distance of 13.5 Angstroms. The atomic coordinates were recorded every 5000 femtoseconds.

### 4.2. Comparison of the Domain–Domain and E6–Domain Interfaces in the MD Simulations of SHP2-wt and SHP2-ΔE6

The domain–domain and E6–domain interfaces in the self-inhibited SHP2 crystal structure and in the MD snapshots of SHP2-wt and SHP2-ΔE6 were identified by determining all residues in a region (domain or E6) falling within a 5-Å threshold from a distinct region. For each region, the distances of the interfacial residues from the closest heavy atom in the interacting region partner were recorded and represented in the figures by heat maps and molecular surfaces with distance-based colors. The linkers between N-SH2 and C-SH2 (a.a. 104–110), between C-SH2 and E6 in SHP2-wt (Gln 214), and between C-SH2 and PTP in SHP2-ΔE6 (Thr253) were not included in the interfacial residues.

### 4.3. N-SH2/PTP Area of Contact in the MD Simulations of SHP2-wt and SHP2-ΔE6

The interfacial area between the N-SH2 and PTP domains was calculated as follows:

$$A(i)=\frac{A{\left(i\right)}_{N-SH2}+A{\left(i\right)}_{PTP}-A{\left(i\right)}_{N-SH2/PTP}}{2}$$

where *A*(*i*)*_N-SH2_*, *A*(*i*)*_PTP_*, and *A*(*i*)*_N-SH2/PTP_* represent, respectively, the van der Waals surface areas of the isolated *N-SH2* domain, isolated PTP domain, and the isolated intramolecular *N-SH2*/*PTP* complex at simulation time *i*.

### 4.4. Volume of the N-SH2 pY Peptide-Binding Cleft in the MD Simulations

The pY peptide-binding cleft in the N-SH2 domain was identified in the groove lining between the BG and EF loops. The volume of this groove was calculated with Swiss-PdbViewer [43] on the MD conformers of N-SH2_closed_, SHP2-wt (triplicate MD runs), SHP2-W248A, SHP2-W248A/E252A, SHP2-ΔE6, and N-SH2_open_ recorded every 1 ns from 3 ns (ca. the time to achieve equilibration) to 30 ns.

### 4.5. Identification of the MD Conformers Showing Lys55/Tyr66 Cation–Pi Interaction

The MD conformers presenting a cation–pi interaction between Lys55 and Tyr66 were identified based on the geometric conditions L ≤ 8 Å and θ ≤ 60° (Figure 7), where L represents the distance between the positive electric charge (lysine N^ζ^ atom) and the π cloud center (tyrosine aromatic ring center) and θ is the angle between the normal to the plane of the aromatic ring and the segment connecting the positive charge with the center of the aromatic ring. The fractions of the conformers presenting the Lys55/Tyr66 cation–pi interaction were calculated over the MD snapshots (recorded every 5000 femtoseconds) in the 3–30-ns time interval of the simulations.

## 5. Conclusions

This study unravels an allosteric mechanism for the stabilization of the closed conformation in the N-SH2 domain of the self-inhibited SHP2 tyrosine phosphatase. The extra-domain E6 region has a key role by interacting with the 34–40 loop and modulating the configuration of critical N-SH2 domain residues. Weakening or abrogating the interactions of E6 with the 34–40 loop by introducing missense mutations, such as Trp248Ala and more plainly Trp248Ala/Glu252Ala or deleting E6, increases the propensity of the N-SH2 domain to undergo closed-to-open conformation change. Given the stability of the closed and open conformations in the isolated N-SH2 domain, these results support the idea that the conversion to the open conformation precedes the undocking of this domain from the PTP domain.

It is important to know the structural determinants of the closed conformation since it delays the binding of pY peptides on the N-SH2 domain, thus cooperating with self-inhibition in the negative regulation of the enzyme. E6 conservation in phosphatases sharing the same tandem SH2 domain architecture suggests that this module is adapted to tune N-SH2 conformation to confer these enzymes with enhanced versatility of regulation and transport across cellular compartments.

Further studies of the complex functions of this enzyme, in particular to gather useful information on the conformational sampling of domains and their interactions with ligands, may take advantage of MD simulations followed by free energy analysis [44,45].

## Figures and Tables

**Table 1:** Summary of the main features characterizing the various structural contexts of the N-SH2 domain in the constructs explored by MD simulations.

Construct	N-SH2 Initial Conformation	N-SH2 Docked onto PTP	34–40 Loop Interactions with E6	34–40 Loop Interactions with PTP	D’E loop Interactions with PTP
SHP2-wt	closed	yes	yes	Yes	yes
SHP2-W248A	closed	yes	weakened *	Yes	yes
SHP2-W248A/E252A	closed	yes	weakened *	Yes	yes
SHP2-ΔE6	closed	yes	no	Yes	yes
SH2_closed_	closed	no	no	no	no
N-SH2_open_	open	no	no	no	no

* Single-missense W248A and double-missense W248A/E252A mutation (Trp248 and Glu252 are E6 residues interacting with N-SH2; see Figure 1 and Figure 2).

## Data Availability

Not applicable.

## Supplementary Materials

The following supporting information can be downloaded at https://www.mdpi.com/article/10.3390/ijms23094794/s1.
