# Supplementary material for: Intramolecular Interaction with the E6 Region Stabilizes the Closed Conformation of the N-SH2 Domain and Concurs with the Self-Inhibitory Docking in Downregulating the Activity of the SHP2 Tyrosine Phosphatase: A Molecular Dynamics Study
Source: Int J Mol Sci. 2022 Apr 27;23(9):4794. doi: 10.3390/ijms23094794 (PMC9105505; doi:10.3390/ijms23094794)
Supplement: Supplementary file 1 [file ijms-23-04794-s001.zip › ijms-1705819-supplementary.pdf]

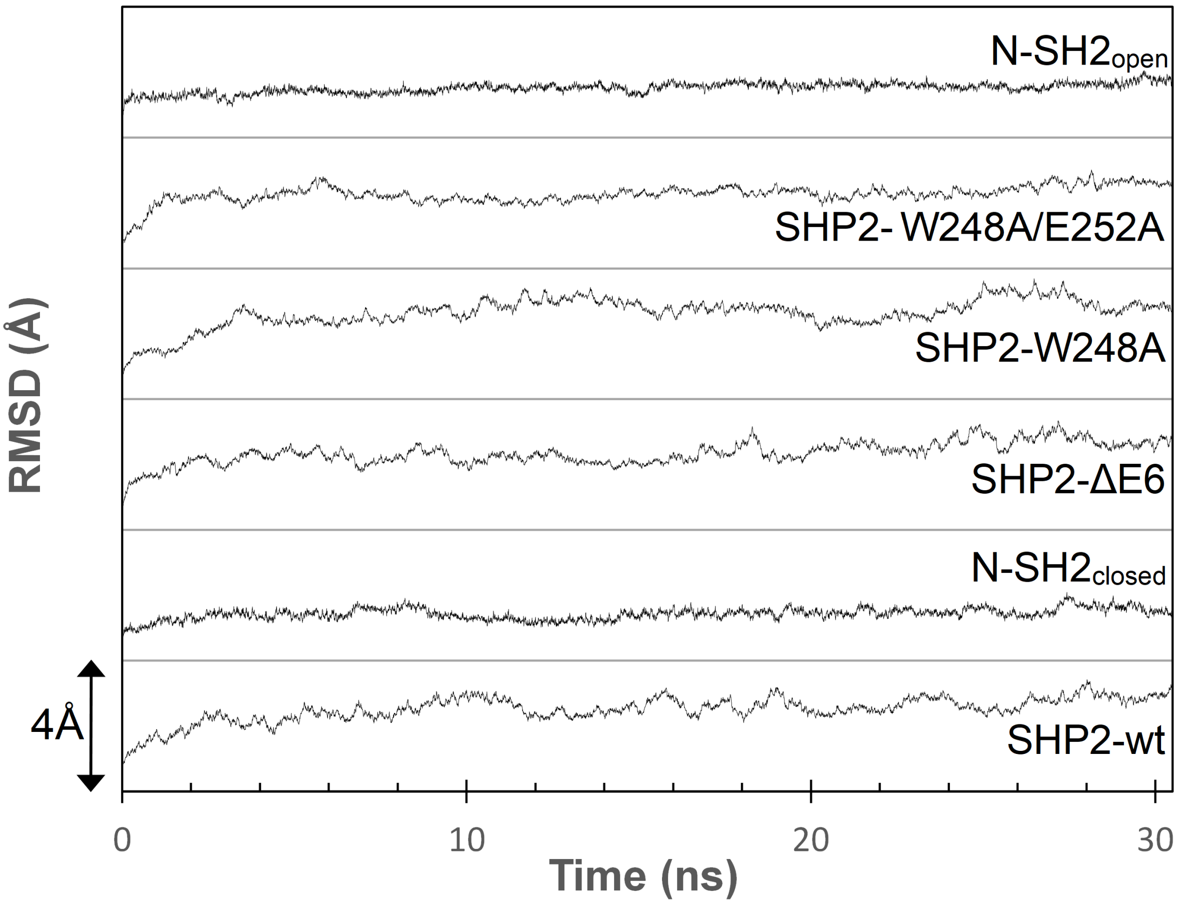

Figure S1. RMSD traces of the MD simulations. RMSDs calculated on backbone atoms after the conformer were aligned to the initial structures employed in the respective MD simulations.

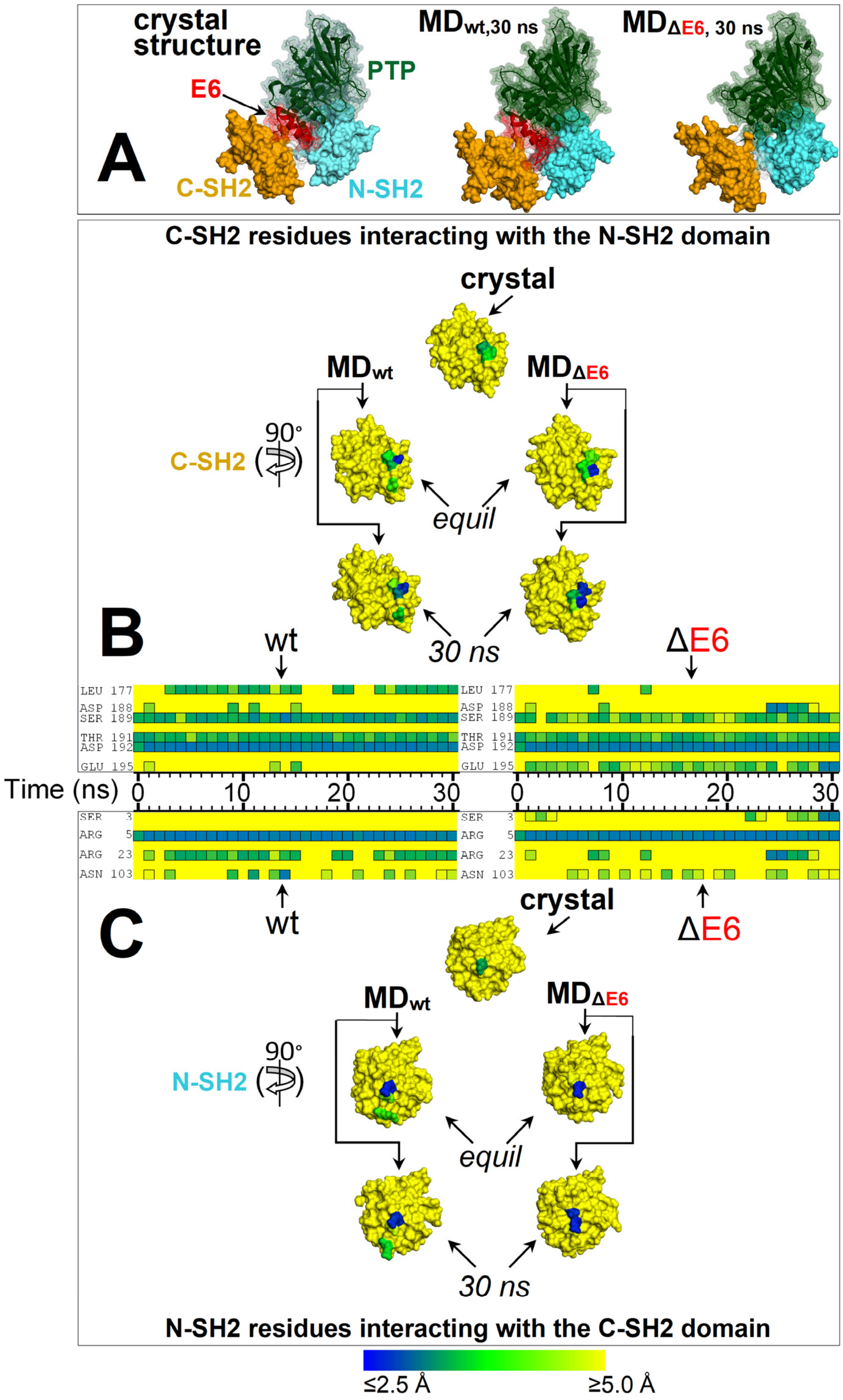

Figure S2. Residues forming the interface between N-SH2 and C-SH2 in SHP2 crystal structure and at time intervals of the MD simulations of SHP2-wt and SHP2-ΔE6. (A) crystal structure of SHP2 (PDB 2SHP) and MD conformers of SHP2-wt and SHP2-ΔE6 obtained at 30 ns. (B) Heat maps and molecular surfaces with the color encoded minimal distances of C-SH2 residues from the N-SH2 domain. (C) Heat maps and molecular surfaces with the color encoded minimal distances of N-SH2 residues from the C-SH2 domain. The molecular surfaces of N-SH2 and C-SH2 have been represented on the domains isolated from SHP2 crystal structure and from MD conformers of SHP2-wt and SHP2-ΔE6 obtained at 3 ns (*ca.* the time to achieve equilibration as assessed from RMSDs) and at 30 ns. The molecular surfaces are rotated by 90° (as indicated by the arrow) with respect to the corresponding domains in panel A to obtain frontal views of the relevant interacting regions. Abscissa in the heat maps represent the MD simulation time (time = 0 represents the minimal distances calculated in SHP2 crystal structure). The distance color key for the heat maps and surfaces of the isolated domains is shown at the bottom.

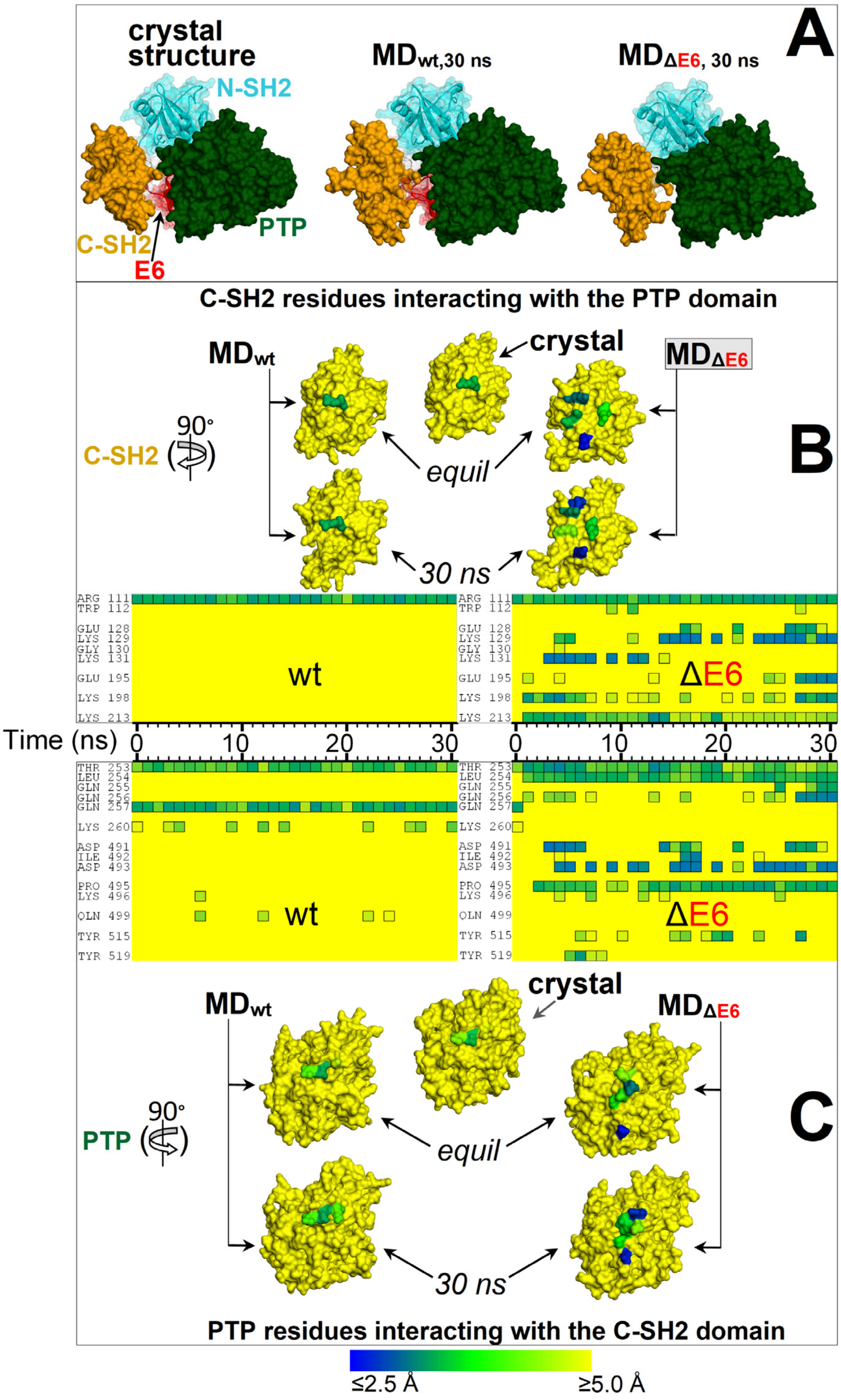

Figure S3. Residues forming the interface between C-SH2 and PTP in SHP2 crystal structure and at time intervals of the MD simulations of SHP2-wt and SHP2-ΔE6. (A) crystal structure of SHP2 (PDB 2SHP) and MD conformers of SHP2-wt and SHP2-ΔE6 obtained at 30 ns. (B) Heat maps and molecular surfaces with the color encoded minimal distances of C-SH2 residues from the PTP domain. (C) Heat maps and molecular surfaces with the color encoded minimal distances of PTP residues from the C-SH2 domain. The molecular surfaces of C-SH2 and PTP have been represented on the domains isolated from SHP2 crystal structure and from MD conformers of SHP2-wt and SHP2-ΔE6 obtained at 3 ns (*ca.* the time to achieve equilibration as assessed from RMSDs) and at 30 ns. The molecular surfaces are rotated by 90° (as indicated by the arrow) with respect to the corresponding domains in panel A to obtain frontal views of the relevant interacting regions. Abscissa in the heat maps represent the MD simulation time (time = 0 represents the minimal distances calculated in SHP2 crystal structure). The distance color key for the heat maps and surfaces of the isolated domains is shown at the bottom.

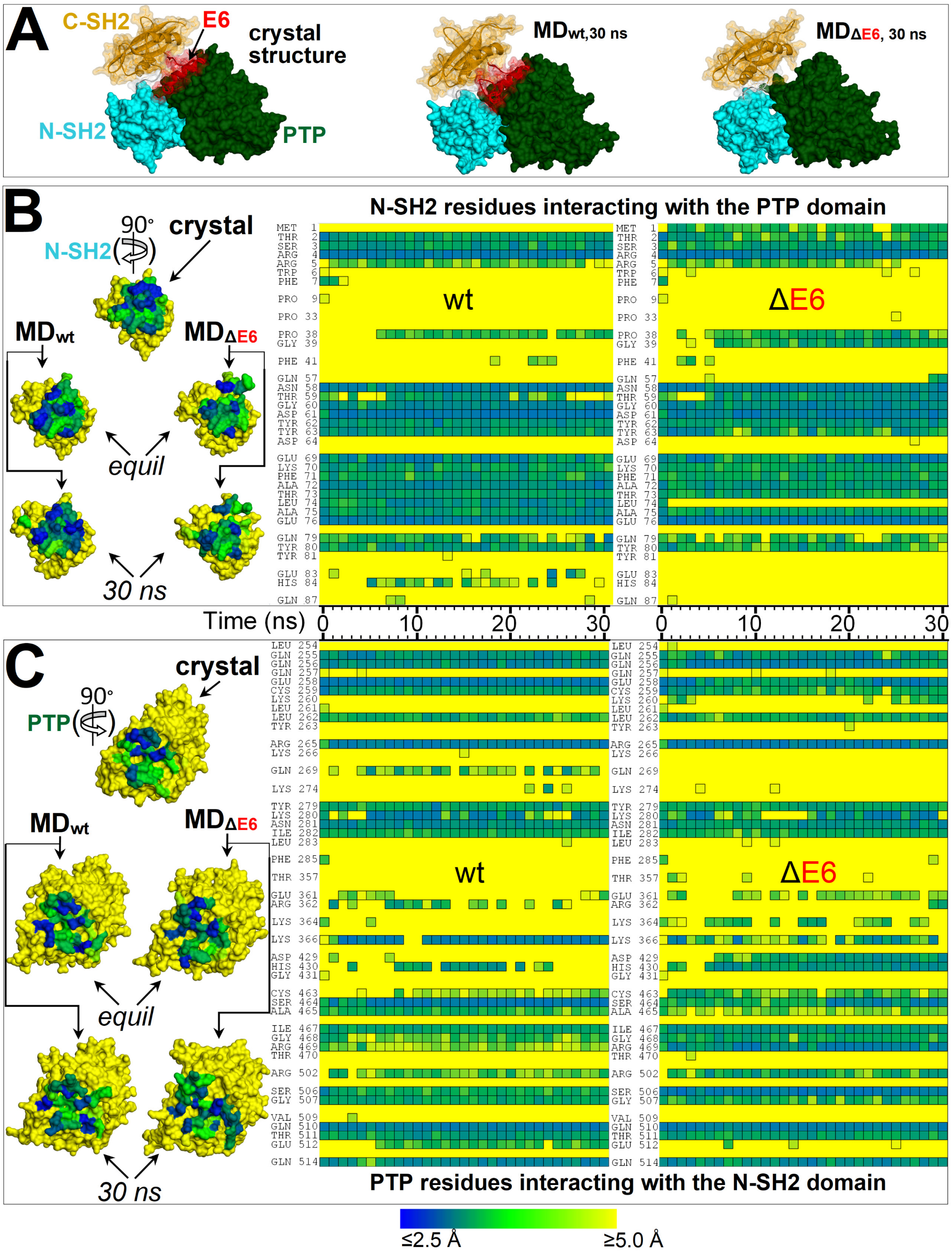

Figure S4. Residues forming the interface between N-SH2 and PTP in SHP2 crystal structure and at time intervals of the MD simulations of SHP2-wt and SHP2-ΔE6. (A) crystal structure of SHP2 (PDB 2SHP) and MD conformers of SHP2-wt and SHP2-ΔE6 obtained at 30 ns. (B) Heat maps and molecular surfaces with the color encoded minimal distances of N-SH2 residues from the PTP domain. (C) Heat maps and molecular surfaces with the color encoded minimal distances of PTP residues from the N-SH2 domain. The molecular surfaces of N-SH2 and PTP have been represented on the domains isolated from SHP2 crystal structure and from MD conformers of SHP2-wt and SHP2-ΔE6 obtained at 3 ns (*ca.* the time to achieve equilibration as assessed from RMSDs) and at 30 ns. The molecular surfaces are rotated by 90° (as indicated by the arrow) with respect to the corresponding domains in panel A to obtain frontal views of the relevant interacting regions. Abscissa in the heat maps represent the MD simulation time (time = 0 represents the minimal distances calculated in SHP2 crystal structure). The distance color key for the heat maps and surfaces of the isolated domains is shown at the bottom.

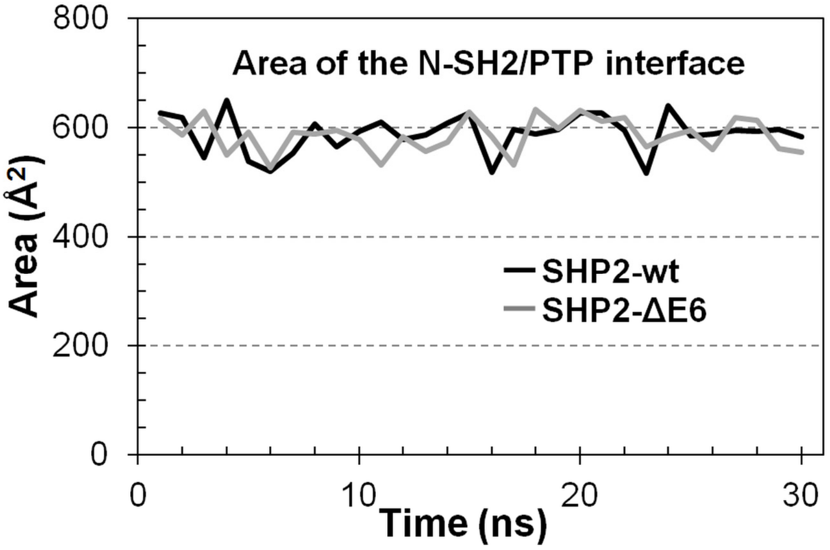

Figure S5. N-SH2/PTP area of contact at time intervals of the MD simulations of SHP2-wt and SHP2-ΔE6.

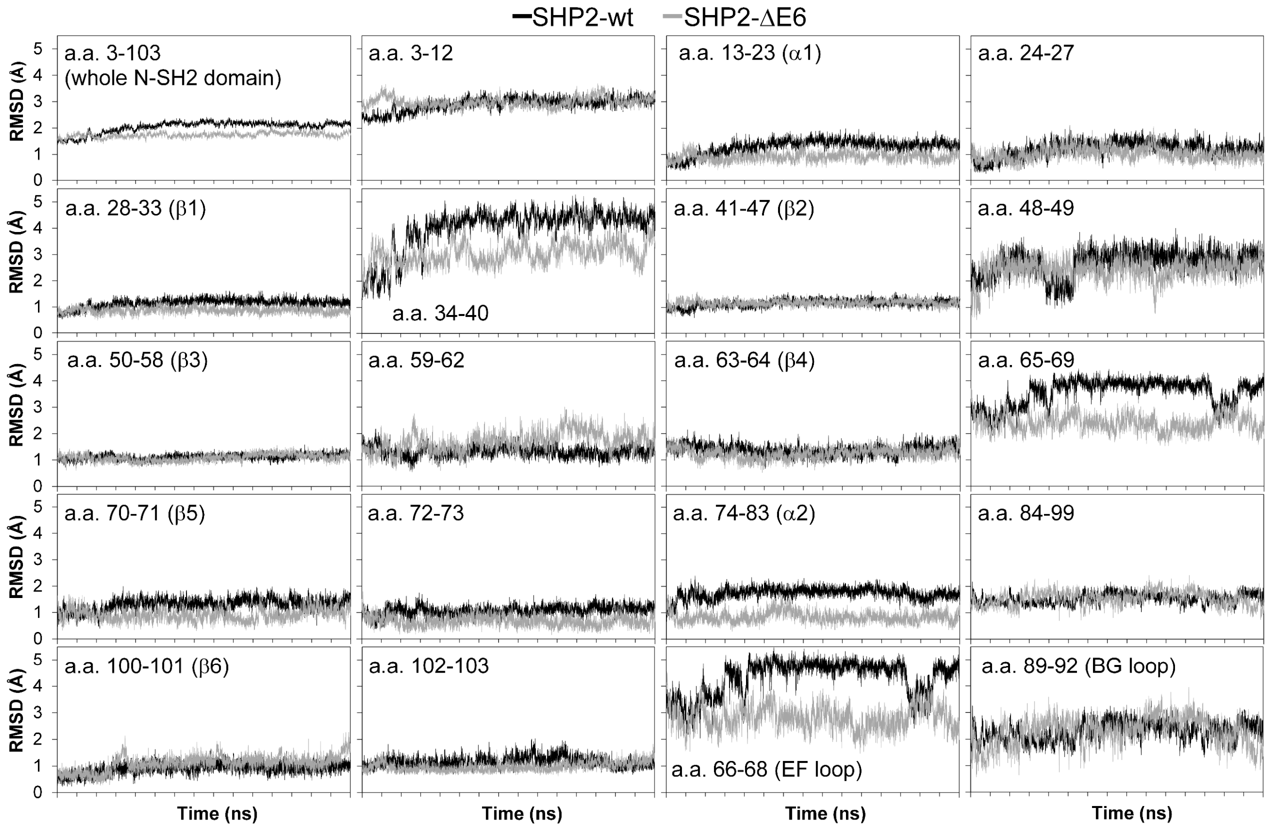

Figure S6. Comparison of the RMSDs of the N-SH2 domain from the MD simulations of SHP2-wt and SHP2-ΔE6. RMSDs were calculated using as the reference the crystal structure of the isolated N-SH2 domain in open conformation (PDB 1AYA, chain A). MD conformers were previously aligned to the same reference across N-SH2 domain residues 3-103 at backbone atoms. RMSDs of the whole N-SH2 domain and of individual secondary structure elements are indicated.

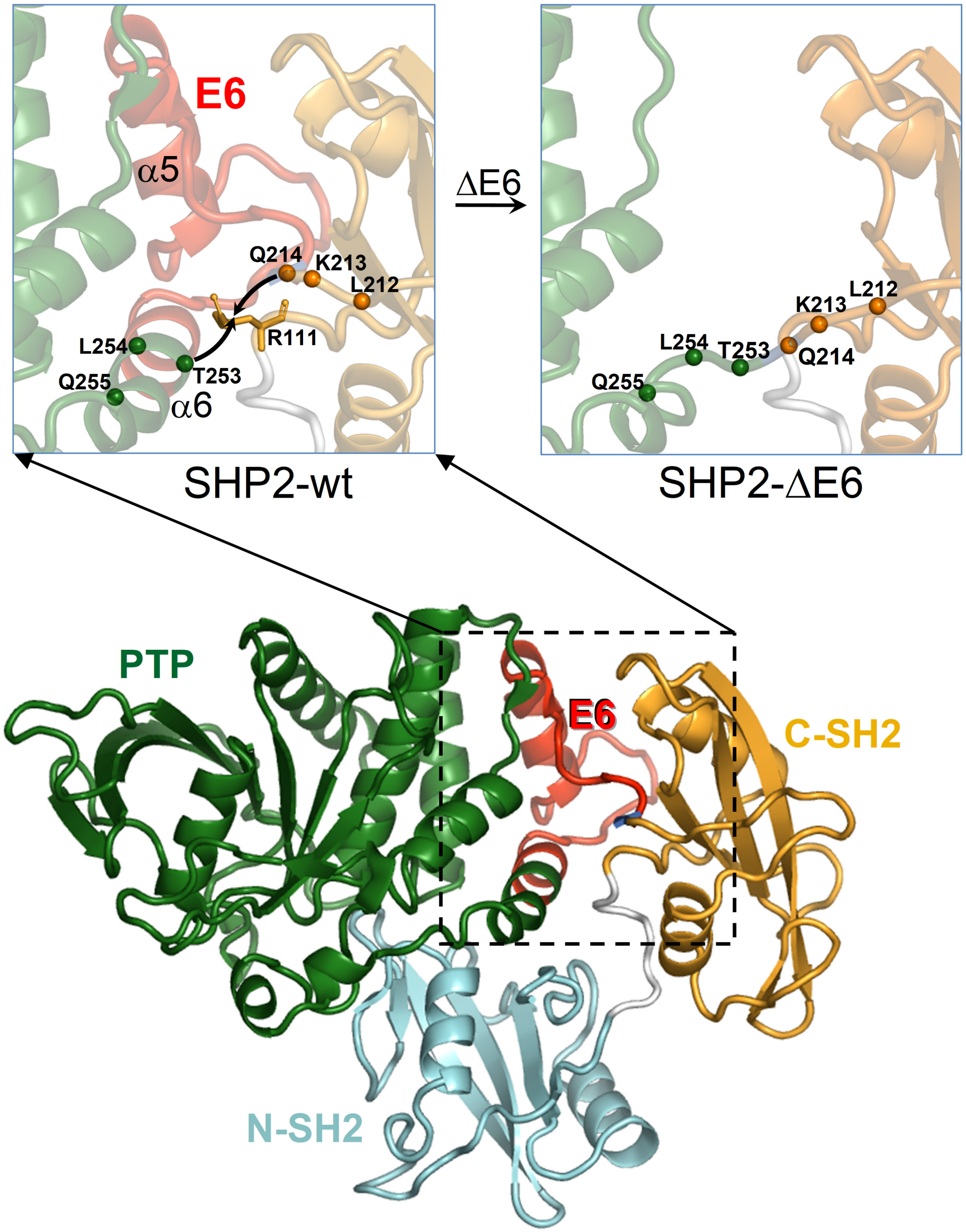

Figure S7. Modelling of E6 deletion construct SHP2-ΔE6 from SHP2-wt. The E6 deletion construct was obtained by removing E6 residues (a.a. 215-252, red ribbons) from SHP2-wt and by linking and optimizing residues 212-214 and 253-255 (C^{α} atoms shown as spheres in same colour as the parent domains). Arg111 (orange sticks) side chain was also optimized as it laid in the path to covalently conjugate the deletion boundaries (indicated by arrows). Please see Materials and Methods for more details.
